# Genetic variation of ITGB3 is associated with Autism Spectrum Disorders (ASD) in South Indian children

**DOI:** 10.1186/1755-8166-7-S1-P109

**Published:** 2014-01-21

**Authors:** Femina KMB Nair, PA Suresh, Moinak Banerjee

**Affiliations:** 1 Human Molecular Genetics laboratory, Rajiv Gandhi centre for Biotechnology, Thiruvananthapura, India; 2 ICCONS, Shoranur, India

## Background

Autism spectrum disorders (ASDs) are a group of developmental disabilities that can cause significant social, communication and behavioral challenges in children. Genetic factors contribute significantly to ASD. ITGB3 encodes integrin β3. This cell adhesion molecule has been implicated as a modulator of serotonergic systems as well as in regulation of synaptic plasticity and maturation. In the brain, integrin β3 couples to integrin αv to form a functional receptor, making integrin αvβ3 an interesting target for regulation of neural 5-HT systems. The aim of this study was to investigate the potential associations of single-nucleotide polymorphisms (SNPs) of the integrin gene with Autism Spectrum Disorder (ASD).

## Material and methods

Hundred and twenty five patients with ASD and 210 healthy volunteers were recruited. Four SNPs of Integrin genes were analyzed by direct sequencing and polymerase chain reaction–restriction fragment length polymorphism genotyping.

## Results

We detected significant allelic and genotypic associations with rs3809865 (Allelic and genotypic p value = 0.0089, 0.0044). Haplotypic association involving risk allele was observed in two, three and four locus. This 3'UTR SNP would decrease/break or enhance/create miRNA-mRNA binding sites and thus affect the expression of host genes in the brain.

## Conclusions

Our finding identified the possible function of this SNP locus, and provides the basis for subsequent functional research.

